# Jianpi Qushi Heluo Formula alleviates renal damages in Passive Hemann nephritis in rats by upregulating Parkin-mediated mitochondrial autophagy

**DOI:** 10.1038/s41598-021-97137-2

**Published:** 2021-09-15

**Authors:** Xin-hui Wang, Rui Lang, Qin Zeng, Ying Liang, Nan Chen, Zhi-zhong Ma, Ren-huan Yu

**Affiliations:** 1 China Department of Nephrology, Xiyuan Hospital of China Academy of Chinese Medical Sciences, Beijing, 100091 China; 2 Graduate School of Chinese Academy of Chinese Medical Sciences, Beijing, 100700 China; 3 Department of Integration of Chinese and Western Medicine, School of Basic Medical Sciences, Peking University, Beijing, 100191 China

**Keywords:** Kidney diseases, Glomerular diseases, Membranous nephropathy

## Abstract

Jianpi Qushi Heluo Formula (JQHF) is an empirical traditional Chinese medicine prescription for treating Membranous Nephropathy (MN) clinically in China. The therapeutic effect of JQHF has been reported in our previous studies. However, the exact mechanism is still unknown. In this study, by establishing an experimental rat model of MN induced by Sheep anti-rat Fx1A serum, we evaluated the effects of JQHF and Tetrandrine (TET), and Benazepril was used as a positive control. As an autophagy agonist, TET is one of the most active components in JQHF. After 4 weeks, significant kidney damage was observed in the rats in the Model group; comparatively, JQHF markedly decreased 24 h urinary protein, Total Cholesterol (TC), and increased serum total Albumin (ALB). Histology showed that JQHF caused significant improvements in glomerular hyperplasia, renal tubular damage, IgG immune complex deposition, and the ultrastructure of mitochondria in MN rats. Flow cytometry analysis showed that treatment with JQHF reduced the level of reactive oxygen species and apoptosis rate, and upregulated mitochondrial membrane potential. Western blot analysis demonstrated that JQHF could protect against mitochondrial dysfunction and apoptosis by upregulating the expression of PINK1, Mitochondrial Parkin, and LC3-II/I, downregulating the expression of Cytoplasmic Parkin, P62, Cytochrome c, and Caspase-3 in the kidneys of MN rats. From images of co-immunofluorescence, it is observed significantly increase in the co-localization of PINK1 and Parkin, as well as LC3 and mitochondria. Similarly, TET treatment significantly upregulated the mitochondrial autophagy and reduced apoptosis in rats after 4 weeks compared with the model group. Comparatively, the ability of JQHF to alleviate renal damage was significantly higher than those of Benazepril and TET. It was demonstrated that JQHF could delay pathology damage to the kidney and hold back from the progression of MN by inhibiting apoptosis and upregulating the mitochondrial autophagy by PINK1/Parkin pathways.

## Introduction

As the main pathological form of adult nephrotic syndrome, Membranous Nephropathy (MN) accounts for about 24.1% of all primary glomerular diseases. Within 10 years, approximately 33–50% of the MN patients will develop end-stage renal disease^1,2^. Disappointingly, there are no safe and effective treatments that slow down the progression of MN. The current orthodox treatment of MN mainly includes immunosuppressive and cytotoxic drugs, which are associated with many disadvantages, low tolerance, and the fact that withdrawal can readily result in recurrence. These factors limit the clinical application of these drugs^3,4^.

Traditional Chinese Medicine (TCM) has accumulated many clinical experience in treating various kinds of kidney diseases, including MN^5^. Among them, the most usual TCM preparations, Jianpi Qushi Heluo Formula (JQHF), was first developed to treat MN by the Department of Nephropathy of Xiyuan Hospital, Chinese Academy of Traditional Chinese Medicine. JQHF contains 10 herbs that Professor Renhuan Yu selected. Professor Yu has treated MN patients with TCM for more than 20 years and has accumulated much theoretical and clinical experience. A clinical study reported that for patients with refractory MN, after a treatment period of 6 months by JQHF, the clinical remission rate was 80% and without apparent side effects^6^. However, the exact mechanism of JQHF on the progression of MN is still unknown.

The pathogenesis of MN is the deposition of immune complexes on the extra capillary sides of the Glomerular Basement Membrane, and resulting in podocytes injury^7^. The deposited immune complexes activate the complement system and subsequently produce the complement membrane attack complex (C5b-9) to insert into the podocyte membranes, and this process is accompanied by the generation of Reactive Oxygen Species (ROS)^8^. The imbalance between ROS production and antioxidant systems will induce oxidative stress. Oxidative stress can attack mitochondrial membranes, cause mitochondrial damage, which could finally lead to cell death^9^. The primary signal transduction pathway includes dissipation of mitochondrial membrane potential (MMP), Cytochrome c (Cyt c) release from mitochondria to the cytoplasm, initiates the apoptosome formation, and finally triggered endogenous caspase apoptosis pathway^10,11^. Intracellularly, mitochondrial-selective autophagy is critical for removing damaged mitochondria and maintaining cellular homeostasis^12^.

Previous studies have demonstrated that mitochondrial dysfunction is strongly linked to renal injury in many experimental models, such as Diabetic Nephropathy (DN), Minimal Change Disease (MCD), and Focal Segmental Glomerulosclerosis (FSGS)^13,14^. Moreover, in these kidney diseases, mitochondrial-targeted therapeutics have been confirmed to help prevent kidney pathogenesis and disease progression. Thus, we hypothesize that accumulated ROS also mediates mitochondrial dysfunction in MN. Activation of mitochondrial autophagy might improve mitochondrial dysfunction and alleviating the apoptosis of kidney cells. Passive Heymann Nephritis (PHN) rat shows pathogenesis similar to MN in humans and has been widely used for the experimental study of MN. In this study, we used PHN rats to evaluate the association between mitochondrial dysfunction and renal injury. Further, we prove that JQHF increases resistance to mitochondrial dysfunction and apoptosis by activated mitochondrial autophagy.

## Results

### JQHF improved the biochemical parameters of PHN rats

**Figure 1:**
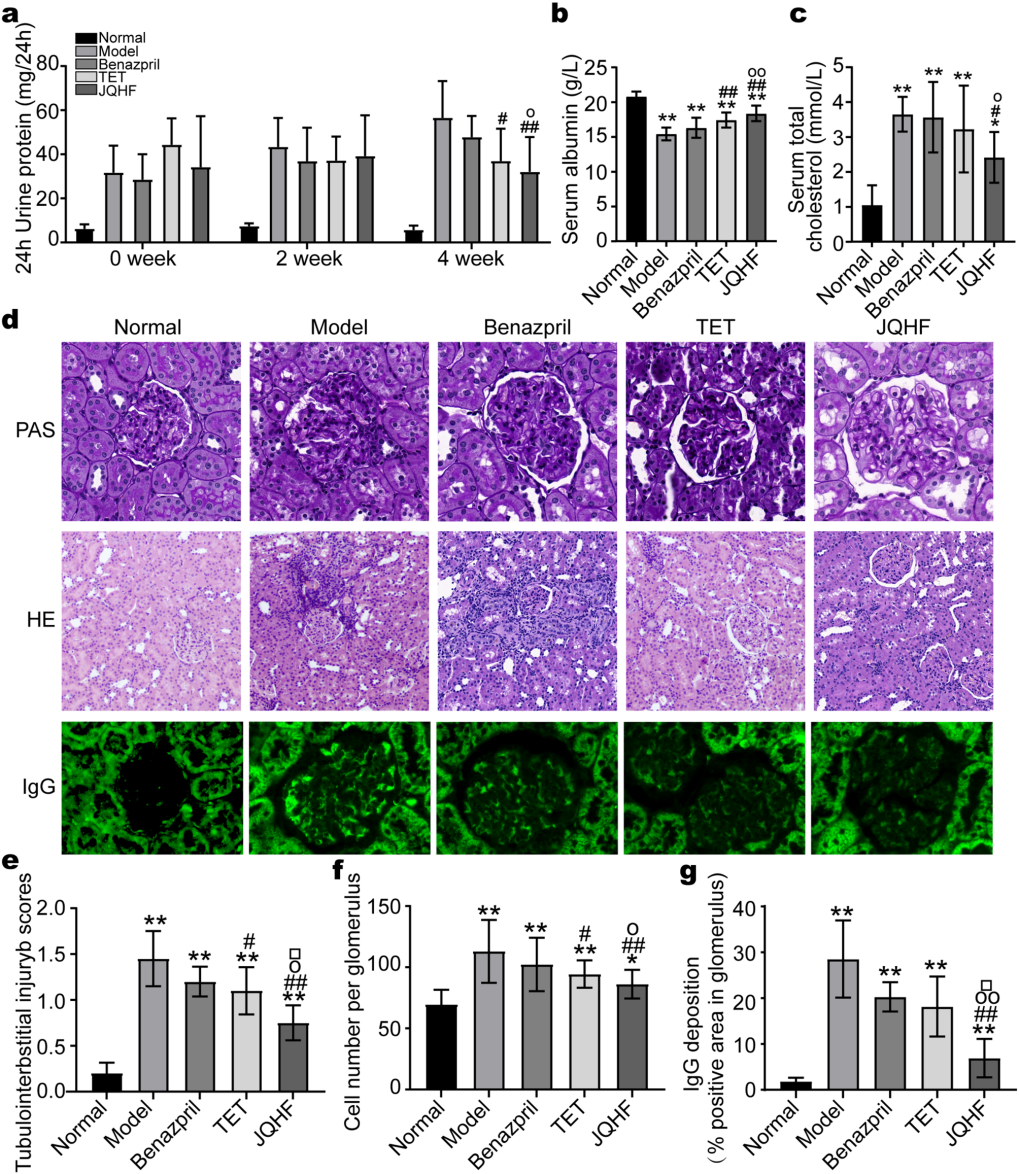
Effects of JQHF on biochemical parameters and renal pathology in PHN rats. (**a**) 24 h urinary protein. (**b**) ALB. (**c**) TC. (**d**) Representative microstructural images of diferent groups. (HE staining original magnifcation  × 100: PAS and IgG staining, original magnifcation  × 400). (**e**) Tubulointerstitial injury scores, (**f**) Glomerular hypercellularity. (**g**) The positive area of IgG deposition in glomerulus. Data were analysed by one-way ANOVA followed by post hoc LSD test (**a**–**c**,**e**–**g**) and presented as the mean ± SD (n = 6). *P < 0.05 vs. Normal group, **P < 0.01 vs. Normal group, ^#^P < 0.05 vs. Model group, ^##^P < 0.01 vs. Model group, ^○^P < 0.05 vs. Benazepril group and ^○○^P < 0.01 vs. Benazepril group.

As shown in Fig. 1a–c, compared with the normal rats, the model rats produced high levels of Total Cholesterol (TC) and 24 h urinary protein (24 h UTP). In contrast, while the level of serum total Albumin (ALB) was significantly reduced. After experimental treatment for 4 weeks, these biochemical parameters were alleviated by the treatment of JQHF. In addition, treatment with JQHF was better than with Benazepril. Similarly, Tetrandrine (TET) treatment significantly reduced 24 h UTP and increased ALB after 4 weeks compared with the model rats. However, there were no significant reductions in TC by TET treatment.

### JQHF reduced renal damage in PHN rats

The results of renal histology and immunofluorescent staining of IgG were revealed by light microscopy (Fig. 1d). The model rats showed the typical pathological feature of MN: glomerular hypertrophy, mesangial cell proliferation, basement membrane thickening, many protein casts in the tubular lumen, some glomerular sclerosis, and IgG deposition along the glomerular capillary wall. Compared with the model group, both the JQHF and TET groups showed significant improvements in glomerular hyperplasia and renal tubular damage. Compared with the TET and Benazepril group, the JQHF group significantly improved renal pathological changes (Fig. 1e,f). Notably, the expressions of IgG also significantly decreased in the JQHF group than in the Model, Benazepril, and TET group (Fig. 1g).

### JQHF protects against mitochondrial dysfunction in the kidney of PHN rats

**Figure 2:**
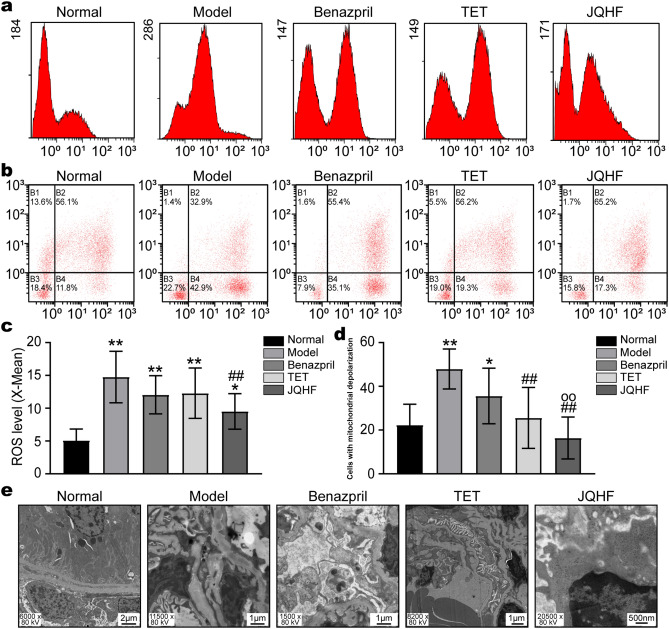
Effects of JQHF on against ROS-induced mitochondrial dysfunction in PHN rats. (**a**) Determination ROS production by DCFA staining. (**b**) Determination of MMP by JC-1 staining. (**c**) Quantitative analysis of intracellular ROS level. (**d**) Quantitative analysis of MMP. (**e**) Analyze mitochondrial ultrastructural injuries by TEM. Data were analysed by one-way ANOVA followed by post hoc LSD test (c and d) and presented as the mean ± SD (n = 6). *P < 0.05 vs. Normal group, **P < 0.01 vs. Normal group, ^##^P < 0.01 vs. Model group and ^○○^P < 0.01 vs. Benazepril group.

According to Fig. 2a–d, compared with the rats in normal groups, ROS levels were elevated, and the MMP levels were decreased in the rats in model groups. After 4 weeks of treatment, as compared with the rats in the model group, we observed a reduction in ROS levels and elevated MMP levels in the JQHF group. Similar results were observed in the TET group, MMP levels were elevated compared to the Model group after 4 weeks.

We used the Transmission Electron Microscope (TEM) to observe the mitochondrial ultrastructural injuries in the kidney of PHN rats. In Normal rats, mitochondria were observed with double membrane structure and transparent cristae, and in model rats, the mitochondria were seen swelled and vacuolized, and the cristae lessened. However, in the rats in JQHF group, the ultrastructural damage of mitochondria was significantly improved, as compared with those rats in the Model, Benazepril, and TET group (Fig. 2e).

### JQHF promotes PINK1/Parkin-mediated mitochondrial autophagy in the kidney of PHN rats

**Figure 3:**
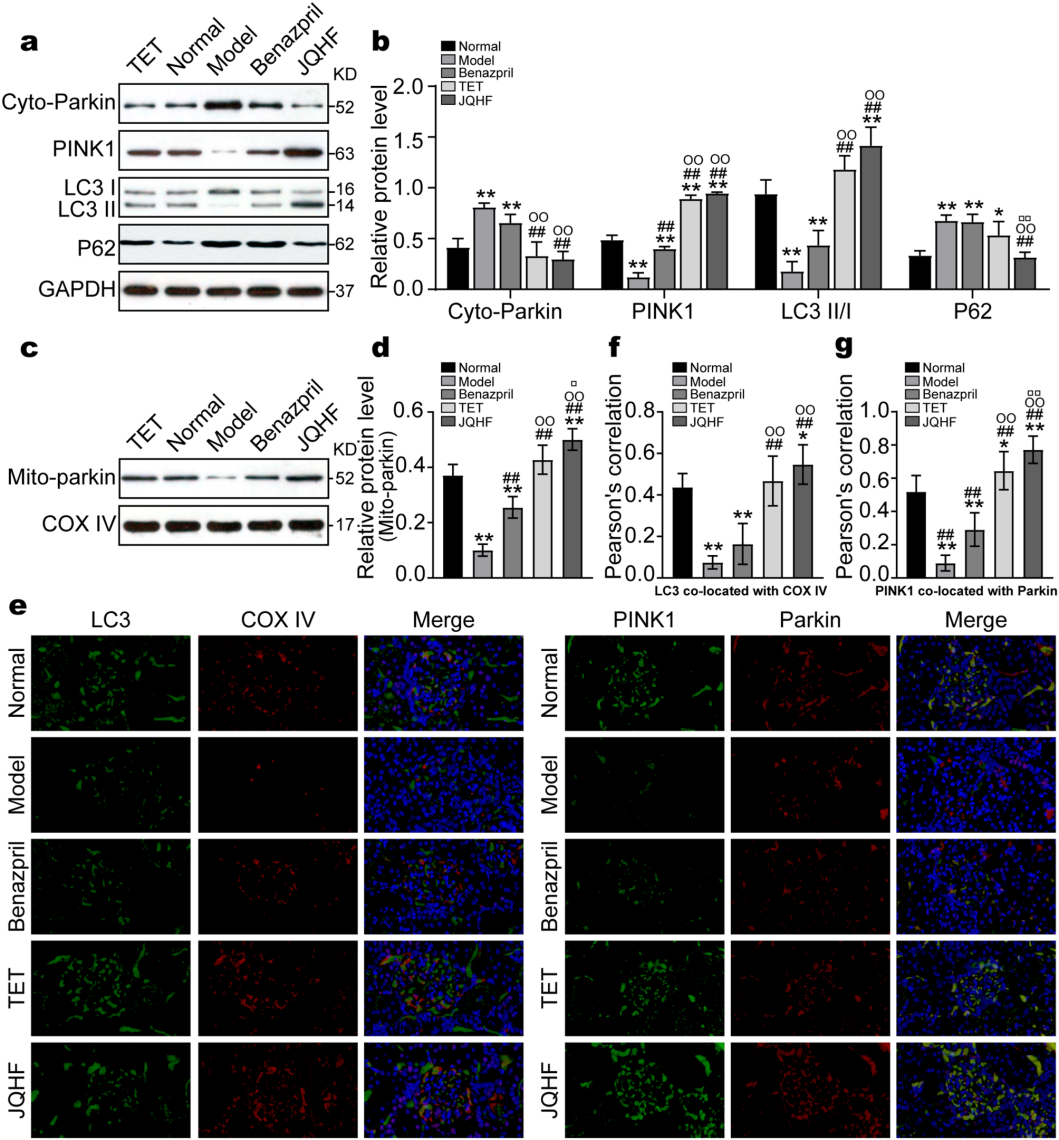
Effects of JQHF on PINK1/Parkin pathway in the kidneys of PHN rats. (**a**,**c**) Western blot analysis of Cyto-parkin, PINK1, LC3, P62 and Mito-Parkin protein expression in each group. Full-length blots are presented in Supplementary Fig. 1 and Supplementary Fig. 4. (**b**,**d**) Quantitative analysis of Cyto-parkin, PINK1, LC3-II/I, P62 and Mito-Parkin protein expression. (**e**) Representative images (×400) of immunofluorescence that LC3 co-localization with COX IV, and PINK1 co-localization with Parkin. COX IV was used as a mitochondrial tracker. LC3 or PINK1 was labeled by fluorescein isothiocyanate (FITC) (green), COX IV or Parkin was labeled by tetramethylrhodamine isothiocyanate (TRITC) (red), and nuclei were stained with DAPI (blue). Strong colocalization signals were seen as yellow dots after merging. (**f**,**g**) Quantitative analysis of the amount of co-localization presented by Pearson’s correlation. Data were analysed by one-way ANOVA followed by post hoc LSD test (**b**,**d**,**f**,**g**) and presented as the mean ± SD (n = 6). *P < 0.05 vs. Normal group, **P < 0.01 vs. Normal group, ^##^P < 0.01 vs. Model group, ^○○^P < 0.01 vs. Benazepril group, ^□^P < 0.05 vs. TET group, and ^□□^P < 0.01 vs. TET group.

We extracted total protein, cytoplasm protein, and mitochondrial protein from renal cortical cells to examine related protein levels. As shown in Fig. 3a–d, the protein levels of PINK1 were reduced significantly in the model rats compared with the normal rats. Meanwhile, the expression of Parkin increased in the cytoplasm, decreased in the mitochondrial, and the conversion of LC3-I to LC3-II was blocked. After 4 weeks of treatment, the expressions of PINK1, Mtio-parkin, Cyto-parkin, and the ratio of LC3-II/I were significantly regulated in the TET and JQHF groups compared to the model and Benzapril groups. Additionally, the results showed that the protein level of P62 was increased in the PHN rats as compared to normal rats, while JQHF treatment significantly lowered the expressions of P62 compared with the model group, TET group, and Benazepril group.

For further verification of the results of the western bolt, we analyze Pearson’s correlation of PINK1 co-located with Parkin and LC3 co-located with mitochondria by immunofluorescence. As shown in Fig. 3e–g, after 4 weeks of treatment, as compared with the model and Benzapril groups, we observed a significant increase in the colocalization of PINK1 and Parkin in the JQHF and TET groups. Furthermore, the colocalization of LC3 and COX IV (mitochondria maker) was also significantly increased in JQHF and TET groups, consistent with the results from the western bolt.

As shown above, both the TET and JQHF treatments significantly activated the mitochondrial autophagy dependent on PINK1/Parkin pathway. Notably, the regulation of JQHF was better than the treatment of TET and Benzapril.

### JQHF blocked the mitochondria-mediated apoptosis in the kidney of PHN rats

**Figure. 4:**
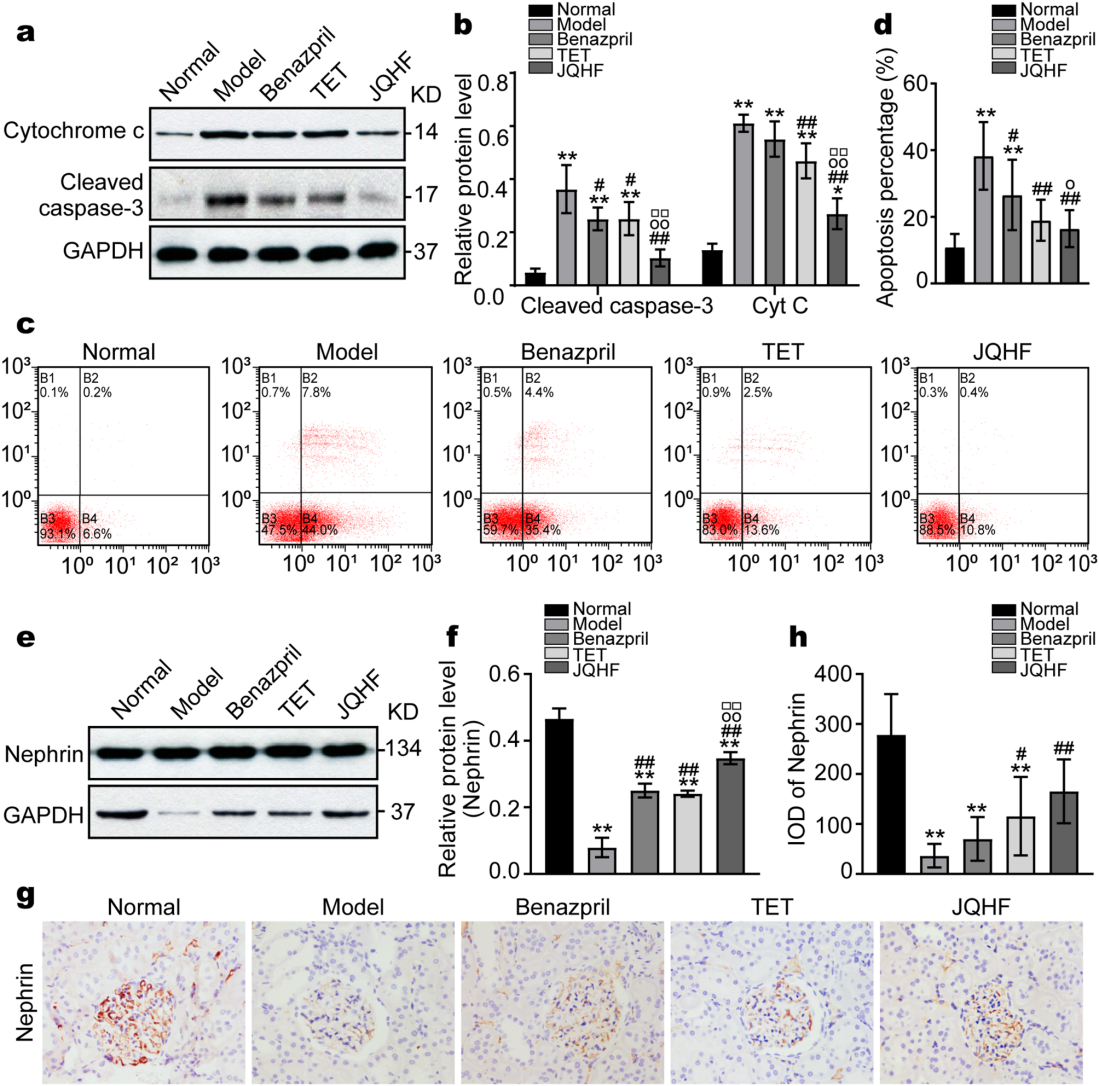
JQHF reduce the apoptosis in the kidneys of PHN rats. (**a**,**e**) Western blot analysis of the expression of Cyt c, Cleaved caspase-3 and Nephrin protein in each group. Full-length blots and all replicates performed are presented in Supplementary Figs. 2, 3. (**b**,**f**) Quantitative analysis of the expression of Cyt c, Cleaved caspase-3 and Nephrin protein. (**c**) Determination of apoptosis rate by Annevin-V-FITC staining. (**d**) Quantifcation of the apoptosis rate in each group. (**g**) Immunohistochemistry staining of Nephrin protein (original magnifcation ×400). (**h**) Analysis of IOD of Nephrin. Data were analysed by one-way ANOVA followed by post hoc LSD test (**b**,**f**,**h**) and presented as the mean ± SD (n = 6). Data were analysed by Kruskal Wallis H test (**d**) (n = 6). *P < 0.05 vs. Normal group, **P < 0.01 vs. Normal group, ^#^P < 0.05 vs. Model group, ^##^P < 0.01 vs. Model group, ^○^P < 0.05 vs. Benazepril group, ^○○^P < 0.01 vs. Benazepril group and ^□□^P < 0.01 vs. TET group.

To determine the effects of JQHF and TET were activating of the mitochondria autophagy on the kidney injury of PHN rats, we initially detection the levels of the apoptosis-promoting proteins released by mitochondria. As shown in Fig. 4a,b, Compared with the normal group, the protein levels of Cyt c and Cleaved caspase-3 were increased in the model group. As compared with the model group, treatment with JQHF and TET significantly decreased the expression of Cyt c and Cleaved caspase-3.

The effect of JQHF and TET on the resistance of apoptosis was further confirmed by the results of annexin-V-FITC/PI staining. The rate of apoptosis in the group model was high as compared with the normal group. However, compared with the model group, treatment with JQHF and TET significantly downregulated the rate of apoptosis in the kidney of PHN rats (Fig. 4c,d).

In addition, podocytes are highly specialized epithelial cells that line the urinary surface of the glomerular capillary tuft in the kidney, and loss of glomerular podocytes is a crucial feature of MN kidney injure. We further investigated the expression of podocyte marker protein Nephrin by western bolt and immunohistochemistry. As shown in Fig. 4e–h, compared with the normal group, the level of Nephrin was decreased in the model group. As compared with the model group, treatment with JQHF and TET significantly increased the expression of Nephrin.

Moreover, taken together these results demonstrate that JQHF and TET reversed apoptosis in PHN rats, and the ability of JQHF to inhibit apoptosis was significantly higher than those of the Benazepril and TET.

## Discussion

JQHF, a traditional Chinese compound formula, has been used to treat MN for several years at Xiyuan hospital. The safety and efficacy of JQHF have been proved by clinical trials. We previously reported that for patients with refractory MN, after a treatment period of 6 months by JQHF, the clinical remission rate was 80% and without apparent side effects^6^. In another study, 30 MN patients were divided into the JQHF and JQHF + Western Medicine (WM) groups. After 6 months of treatment, the levels of 24 h UTP and ALB were improved in both of the groups, and the JQHF group is better than the JQHF + WM group^15^. Our clinical studies results suggested the profound renoprotective effects of JQHF on MN, and next, we tried to explore the kidney protection mechanism of JQHF. This study, induced the PHN model in Male Sprague–Dawley (SD) rats by a single tail vein injection of sheep anti-rat FX1A serum, which has a similar pathogenic mechanism and clinical manifestations to MN in humans^16^. After 4 weeks of treatment, our data indicate that JQHF treatment reduced the levels of 24 h UTP and TC, and increased the level of ALB, which is better than the treatment of Benazepril.

Currently, MN was understood as an autoimmune disease in which IgG autoantibodies form subepithelial immune complexes with autoantigens expressed on the podocyte cell surfaces^17^. In human idiopathic MN, the major autoantigen on the cell surface of podocytes is M-type phospholipase A2 receptor (PLA_2_R) which could be found in 70–80% of patients^18^. In PHN rats, the antigen responsible is the polyspecific receptor Megalin, which cannot be found in human podocytes^19^. Although the major autoantigen of podocytes is different, the processes of the deposited immune complexes that activate podocytes are similar^20^. The deposited immune complexes activate the complement system and subsequently produce C5b-9 to insert into the podocyte membranes, and this process was confirmed could cause the activation of the signaling pathways of NADPH oxidase and accompanied by the generation of ROS^21^.

The production of ROS was considered as an upstream step in oxidative damage to mitochondrial dysfunctions^22^. In recent years, a close relationship between mitochondrial dysfunctions and renal glomerular and tubular diseases has been established^23^. In the FSGS model rats, the copy number of mitochondrial DNA (mtDNA) in glomerular is significantly reduced, suggesting that mitochondrial dysfunction may contribute to the pathogenesis of FSGS^24^. In patients with congenital nephrotic syndrome, the level of mitochondrial cytochrome oxidase I (COX I) in renal tissue was significantly decreased^25^. In vitro, high glucose can induce ROS in podocytes, and the oxidized mtDNA is more potent in activating inflammasomes and apoptosis in macrophages^26^. According to Guan et al., mitochondrial fragmentation participated in podocyte injury in adriamycin nephropathy rats^27^. A case report described a patient who had IgG4-related MN disease, the IgG4 retrieved from the serum of this patient caused mitochondrial dysfunction in cultured podocytes^28^. Nevertheless, there are only a few studies investigating mitophagy in MN kidney injure.

In this study, we observed displayed mitochondrial dysfunctions in the kidney of model rats. As expected, treatment with JQHF downregulated the level of intracellular ROS, improved the level of MMP, and the ultrastructural damage of mitochondria was also significantly improved. These results indicated that mitochondrial dysfunctions correlate with kidney injure in the PHN rats, and JQHF could ameliorate kidney injury via against mitochondrial dysfunction. According to the previous studies, there are at least three central mechanisms through which damaged mitochondria can kill their host, that through the production of ROS induce mutations in mitochondrial DNA (mtDNA), pro-inflammatory signals, or increase the tendency of mitochondria to release proapoptotic proteins (Cyt c) from the intermembrane space into the cytoplasm^29,30^. Autophagy is the only mechanism that occurs via the lysosomal pathway to engulf and recycle the aging and damaged organelles^31^.

In intracellular, PINK1 and Parkin are regarded as mediators to selective degradation of damaged mitochondria by mitochondrial autophagy. Parkin was confirmed to mediate a mitophagy quality control pathway and selectively bind to damaged mitochondria^32^. Recent studies have shown that the process of mitochondrial depolarization enhanced the stability of PINK1, and PINK1/Parkin pathway plays an essential role in maintaining mitochondrial homeostasis by operating as an initiator of mitophagy^33^. A study confirmed that Mitophagy, dependent on PINK1 and Parkin, was activated in renal proximal tubular cells in acute kidney injury (AKI). Mice genetically deletion in PINK1 or Parkin, and deficient in both PINK1/Parkin, were all susceptible to ischemic AKI^34^. Wen et al. reported that PINK1/Parkin pathway activation could individually ameliorate injured podocytes in DN mice^35^.

Autophagy mediated by PINK1/Parkin pathway is occurring accompanied by the increase of PINK1 protein, the recruitment of parkin to the mitochondria, and P62 links damaged mitochondria to LC3-II^36^. Our results confirmed that JQHF treatment increased the expression levels of PINK1, Mito-Parkin, and the ratio of LC3-II/I and decreased Cyto-parkin and P62 protein expression. Further, the immunofluorescence double-labeling technique was used to observe the co-localization of Parkin with PINK1, LC3 with mitophagy. From images and co-localization analysis, we observed a significantly increased in the colocalization of PINK1 and Parkin, as well as LC3 and mitochondria. Among them, we can safely conclude, JQHF could be activated the Pink1/Parkin-dependent mitophagy pathway in the kidney of PHN rats.

We next analyzed the consequences of JQHF regulation mitochondrial autophagy on apoptosis. It was observed that in the model rats, the level of Cyt c, Caspase-3 were upregulated. Cyt c is usually found in the mitochondrial intermembrane space, indicating apoptosis caused by mitochondrial damage in MN rats^37^. The effect of JQHF on the resistance of apoptosis was further confirmed by the results of Annexin-V-FITC/PI staining. Compared with the model group, JQHF treatment decreased the apoptosis rate from 45% to less than 15%. We also observed increased expression of Nephrin protein. Thus, our study suggested that podocyte damage was ameliorated in the rats with JQHF treatment.

TET is a bisbenzylisoquinoline alkaloid isolated from the *Radix* *stephaniae tetrandrae*^38^. Moreover, *Radix* *stephaniae tetrandrae* is one of the most active components in JQHF. Previous studies have confirmed that TET has definite effects on anti-hypertensive, anti-inflammatory, and anti-cognitive impairment^39,40^. Recent research has shown that, compared with valsartan, a low concentration of TET exhibited better effects in reducing the apoptosis mediated by TRPC6 in podocytes^41^. We systematically assessed the therapeutic effect of TET on IMN rats. The results indicated that TET could protect renal function to a certain extent, although it is not as effective as JQHF. In addition, TET treatment significantly upregulates the PINK1/Parkin pathway and decreases apoptosis in rats after 4 weeks compared with the model group.

Nevertheless, surprisingly, the expression levels of the P62 had no remarkable difference between the TET group and the model group. As a critical selective autophagy adaptor protein, p62 can mediate mitochondrial clearance of ubiquitin through Pink1/Parkin signal pathway. Therefore, the increase of P62 level generally indicates that autophagy is inhibited. However, the expression of P62 may also increase when autophagy activation, especially in toxic stimulation and oxidative stress^42,43^. The mechanism may be that the expression level of p62 will be significantly up-regulated under oxidative stress conditions, and this process is mainly regulated by the transcription factor EB. Combined with the results of flow cytometry, the intracellular ROS levels in the TET group also no significant improvement compared with the model group. In PHN rats, the interaction between TET treatment and oxidative stress still needs further research.

In conclusion, our results showed that JQHF treatment attenuated the 24 h UTP and prevented the progression of MN. Further, we confirm that JQHF ameliorated kidney injury via against mitochondrial dysfunction. Meanwhile, JQHF corrects the expression levels of PINK1, Parkin, and LC3 markedly, thereby playing a mitochondrial protection role through PINK1/Parkin pathway. TET also has a therapeutic effect on PHN rats, while the abilities to alleviate renal damage and regulate autophagy by JQHF were significantly higher than that by TET. This study might facilitate the application of JQHF in the prevention and treatment of MN.

## Materials and methods

### Preparation of JQHF

Nine herbs, ingredients of JQHF, were purchased from Hebei Baicaokang Pharmaceutical Co., Ltd. (Hebei, China, Certificate No.JIY20190064). JQHF is composed of *Astragalus membranaceus, Rhizoma atractylodis macrocephala*, *Poria, Radix stephaniae tetrandrae*, *Folium perillae*, *Dioscorea nipponica Makino*, *Panax gingseng*, *Radix angelica sinensis*, and *Folium nelumbinis*. In the clinic, JQHF crude drug content of 180 g is used to making water decoction and oral two times a day to treat patients. This is equivalent to 16.2 g/kg/day in the rats. All herbal drugs were prepared by the Pharmacy Department of Xiyuan Hospital, and the decoction was adjusted to 1.62 g/ml with water for the rats for oral gavage dose.

### Animals and treatment administration

Our animal experiment was conducted under the Arrive Guidelines, national and institutional rules regarding animal experimentation, following pre-approval by the Animal Ethics Committee, Xiyuan Hospital, China’s Academy of Chinese Medical Sciences (Beijing, China, approved No. 2019XLC020-2).

SD rats (150 ± 10 g, 5 weeks) were provided by Beijing Vital River Laboratory Animal Technology Co. Ltd (Beijing, China, Certificate No. SCXK (JING) 2016-0006). The SD rats were housed in a special pathogen-free (SPF) environment, provided by the Experimental Animal Centre of Xiyuan Hospital (Beijing, China, Certificate No.SYXK (JING) 2015-0011).

The rats were fed within a temperature-controlled facility (25 °C, 50% relative humidity) under a 12 h light/dark cycle, and rats were allowed free access to pure water and standard chow. To induce PHN, sheep anti-rat Fx1A serum (PTX-002S, Probetex, San Antonio, USA) was injected into a tail vein of each rat with 0.5 ml/100 g body weight. After 2 weeks, we randomly selected two rats for analysis. The rats were sacrificed, and electron microscopy was used to investigate the deposition of immune complex and diffuse thickening of the basement membranes. These pathological changes indicated the successful induction of the PHN model. Twenty-four PHN rats were randomly divided into four groups. Additionally, six healthy Male SD rats were chosen as normal controls group. For experimental intervention, normal rats were orally treated with water (1 ml/100 g body weight) daily. For the Model group, PHN rats were orally treated with water (1 ml/100 g body weight) daily. For the JQHF group, PHN rats were orally treated with JQHF decoction daily (JQHF orally gavage dosage: 1.62 g/100 g body weight) daily. According to international guidelines, the dosage of rats was determined according to the dosage conversion between patients and animals. In the TET group, PHN rats were given a daily oral gavage dose of 0.5 mg/100 g body weight of TET (20190717, Zelang, Nanjing, China). Finally, Benazepril was used as a positive control. In the Benazepril group, according to the human clinical dose, PHN rats were orally gavage with Benazepril (X3080, Nuohua, Beijing, China) 1 mg/100 g body weight daily. Drug administration was carried out for a total of 4 weeks.

### Measurement of biochemical parameters

Rats were housed in separate metabolic cages every 2 weeks for 24 h urine collection. At the end of 4 weeks of drug administration and rats fasted for 12 h, blood samples were collected from the abdominal aorta. Then, kidney tissue samples were collected immediately after euthanasia by isoflurane inhalation. Serum was separated (3500 r/min, 15 min) for the examination of TC and ALB. All biochemical parameters were detected by the automatic biochemistry analyzer (Ccbas 8000, Roche Diagnostics GmbH, Mannheim, Germany).

### Pathological testing of renal tissue

The left kidneys were immediately put into 10% phosphate-buffered formalin solution; after fixed kidney specimens, the specimens were embedded in paraffin. Next, we observe glomerular and tubulointerstitial damage with hematoxylin–eosin (HE) and periodic acid-Schiff (PAS) stain. The stained samples were assessed by two pathologists by utilizing a Nikon Eclipse Ti-SR (Nikon Corporation, Tokyo, Japan) inverted fluorescence microscope.

To observe glomerular hypercellularity, we examined at least 10 glomeruli in the histological section from each animal. For each group, every section was examined to acquire typical photographs (at a magnification of 100×). We counted the total number of cells in each glomerulus. When achieving photos, we ensured that the background lighting of each picture was consistent and the tissue was occupied the whole field of vision. Image-Pro Plus 6.0 software (Media Cybernetics, Inc., Rockville, USA) was used to select glomeruli in each photograph.

The stained sections of HE were used to evaluate the tubulointerstitial injury. Renal tubular atrophy, dilatation, tubular type, interstitial inflammation, and interstitial fibrosis were evaluated in 10 fields on each renal section at a magnification of × 100. We used a semi-quantitative renal histological grading scale (from 0–3): 0 = normal; 1 = lesions in the < 25% region of the area of the section; 2 = lesions in the 25% to 50% region; 3 = lesions in the > 50% region^44,45^.

### Immunofluorescence staining

Paraffin-embedded Kidney tissues were cut for 3 μm thickness sections for immunofluorescence staining. The sections were baked at 60 °C for 3 h, followed by deparaffnization, hydration, and antigen retrieval. Then, the sections were incubated in 0.25% phosphate-buffered saline (PBS) tween and soaked in 3% bovine serum albumin (BSA) blocking before incubation with anti-rat IgG labeled with FITC (4416s, CST, Boston, USA). The sections were then soaked in PBS in a dark room. The positive area of immune complex deposition in the glomerulus was then quantitated using Image-Pro Plus 6.0 software.

Next, we observe the immunofluorescence that PINK1 co-localization with Parkin, and LC3 co-localization with COX IV. Similarly, after the deparaffnization, hydration, and antigen retrieval, the sections were blocked in 3% BSA for 30 min. Then, Anti-LC3 (2775, CST, Boston, USA) was coupled with Anti-COX IV (6025-1-1 g, Proteintech, Wuhan, China), and Anti-PINK1 (DF7742, Affinity, Ohio, USA) was coupled with Anti-Parkin (SC-32282, Santa, Dallas, USA), and incubated overnight at 4 °C. After washing with 0.25% PBS, the sections were incubated with secondary antibodies for 1 h, and DAPI (Beyotime) was used to visualize the nuclei. Finally, the sections were visualized under an epifluorescence microscope (LSM510-meta, Zeiss, Aalen Germany). The protein co-localization was then quantitated using Image-Pro Plus 6.0 software.

### Immunohistochemistry staining

The kidney sections were deparaffinization, hydration, and antigen retrieval with 10 mM citrate buffer (pH 6.0). Then, 3% BSA blocking and allowed to incubate with primary antibody (Nephrin: ab136894, Abcam, Cambridge, UK). Afterward, the sections were washed with PBS and incubated with appropriate biotinylated secondary antibodies at room temperature, and then the sections were stained with 3,3′-diaminobenzidine (DAB) and hematoxylin respectively. Image-Pro Plus 6.0 software was used to analyze the integral optical density (IOD) of Nephrin in each visual field.

### TEM examination

We cut the other 1 mm^3^ segment of kidney tissue for TEM examination. Kidney tissue was fixed with 2.5% glutaraldehyde and 0.5% osmium tetroxide successively. Afterward, the fixed sections were followed by dehydrated, penetrated with acetone, and embedded with epoxy resin. Then the ultrathin pieces (60–80 nm) were stained with uranyl acetate and lead citrate respectively, and observed using an electron microscope (JEM-1230, JEOL Japan Electronics Co., Ltd, Tokyo, Japan).

### Flow cytometry analysis

We first sacrificed the rats and then immediately dissected fresh kidney tissue. A single-cell suspension from the rat kidney cortex was prepared using gentle mechanical forces, then filtered with a 400-mesh screen with 40 μm and evenly spaced. Finally, the concentration of single-cell suspension was adjusted to 1 × 10^6^/ml. For intracellular ROS, MMP, and cells apoptosis analysis, single-cell suspension was incubated with dichlorofluorescein diacetate (DCFH-DA) (S0033, Beyotime, Jiangsu, China), tetrechloro-tetraethylbenzimidazol carbocyanine iodide (JC-1) (C2006, Beyotime, Jiangsu, China), and Annexin-V-FITC/PI (10010-02, SBA*, Birmingham, USA*) for 30 min, respectively. The cells were then harvested for flow cytometric analysis using Flow Cytometer (EPICS ELITE, Beckman Coulter, Inc., Los Angeles, USA).

### Western blot analysis

The proteins of renal cortex tissue were lysed with Protein Extraction Kit (P0033, Beyotime, Jiangsu, China), the proteins of mitochondria and cytoplasmic were extracted with Tissue Mitochondria Isolation Kit (C3606, Beyotime, Jiangsu, China) and quantified by BCA protein assay kit (P1511, Applygen, Beijing, China). Total tissues lysates were fractionated by sodium dodecyl sulfate–polyacrylamide gel electrophoresis (SDS-PAGE) and then transferred to nitrocellulose membranes. Afterward, the membranes were blocked with 5% BSA for 1 h at room temperature and followed by incubation with the primary antibodies anti-Parkin (SC-32282, Santa, Dallas, USA), anti-PINK1 (DF7742, Affinity, Ohio, USA), anti-LC3 (2775, CST, Boston, USA), SQSTM1/p62 antibody (5114, CST, Boston, USA), anti-Caspase-3 (9662S, CST, Boston, USA), anti-Cyt c (11940S, CST, Boston, USA), and anti-Nephrin (ab136894, Abcam, Cambridge, UK) overnight at 4 ℃. After washing, the corresponding HRPconjugated secondary antibodies were used. Finally, we used an ECL Kit (P1010, Applygen, Beijing, China) and a multi-functional imaging system to detect positive binding. Image-Pro Plus 6.0 software was used to quantify all protein bands.

### Statistical analyses

Statistical analyses were performed using SPSS version 23.0 software (IBM, Inc., Chicago, USA). Data are presented as mean ± standard deviation(SD) unless otherwise indicated. Two-group comparisons were performed using an independent-sample t-test. Multiple-group comparisons were analyzed by One-Way Analysis of Variance (one-way-ANOVA) followed by post hoc LSD test when normality (and homogeneity of variance) assumptions are satisfied; otherwise the Kruskal Wallis H test will be used. P < 0.05 was recognized to be statistically significant.

## Supplementary Information

Supplementary Information.

## Data Availability

All data included in this study are available upon request by contact with the corresponding author.
