# Supplementary material for: Jianpi Qushi Heluo Formula alleviates renal damages in Passive Hemann nephritis in rats by upregulating Parkin-mediated mitochondrial autophagy
Source: Sci Rep. 2021 Sep 15;11:18338. doi: 10.1038/s41598-021-97137-2 (PMC8443625; doi:10.1038/s41598-021-97137-2)
Supplement: Supplementary file 1 — Supplementary Information. [file 41598_2021_97137_MOESM1_ESM.doc]

**Supplementary Information**

**Jianpi Qushi Heluo Formula alleviates renal damages in Passive Hemann nephritis in rats by upregulating Parkin-mediated mitochondrial autophagy**

Xin-hui Wang^1,2,5^, Rui Lang^1,5^, Qin Zeng^1,3^,Nan Chen^1,3^, Zhi-zhong Ma^4,6^* and Ren-huan Yu^1,6^*

^1^China Department of Nephrology, Xiyuan Hospital of China Academy of Chinese Medical Sciences, Beijing, 100091, China.

^2^Graduate School of Chinese Academy of Chinese Medical Sciences, Beijing, 100700, China.

^3^Graduate School of Beijing University of Chinese Medicine, Beijing, 100029, China. ^4^Department of Integration of Chinese and Western Medicine, School of Basic Medical Sciences, Peking University, Beijing, 100191, China.

^5^These authors contributed equally: Xin-hui Wang and Rui Lang.

^6^These authors contributed equally: Zhi-zhong Ma and Ren-huan Yu.

*Correspondence should be addressed to Zhi-Zhong Ma, mazzbumc@sina.com；

Ren-huan Yu, [tezhongeyu@vip.sina.com](mailto:tezhongeyu@vip.sina.com;).

Supplementary Figure 1. Original blots for Figure 3.


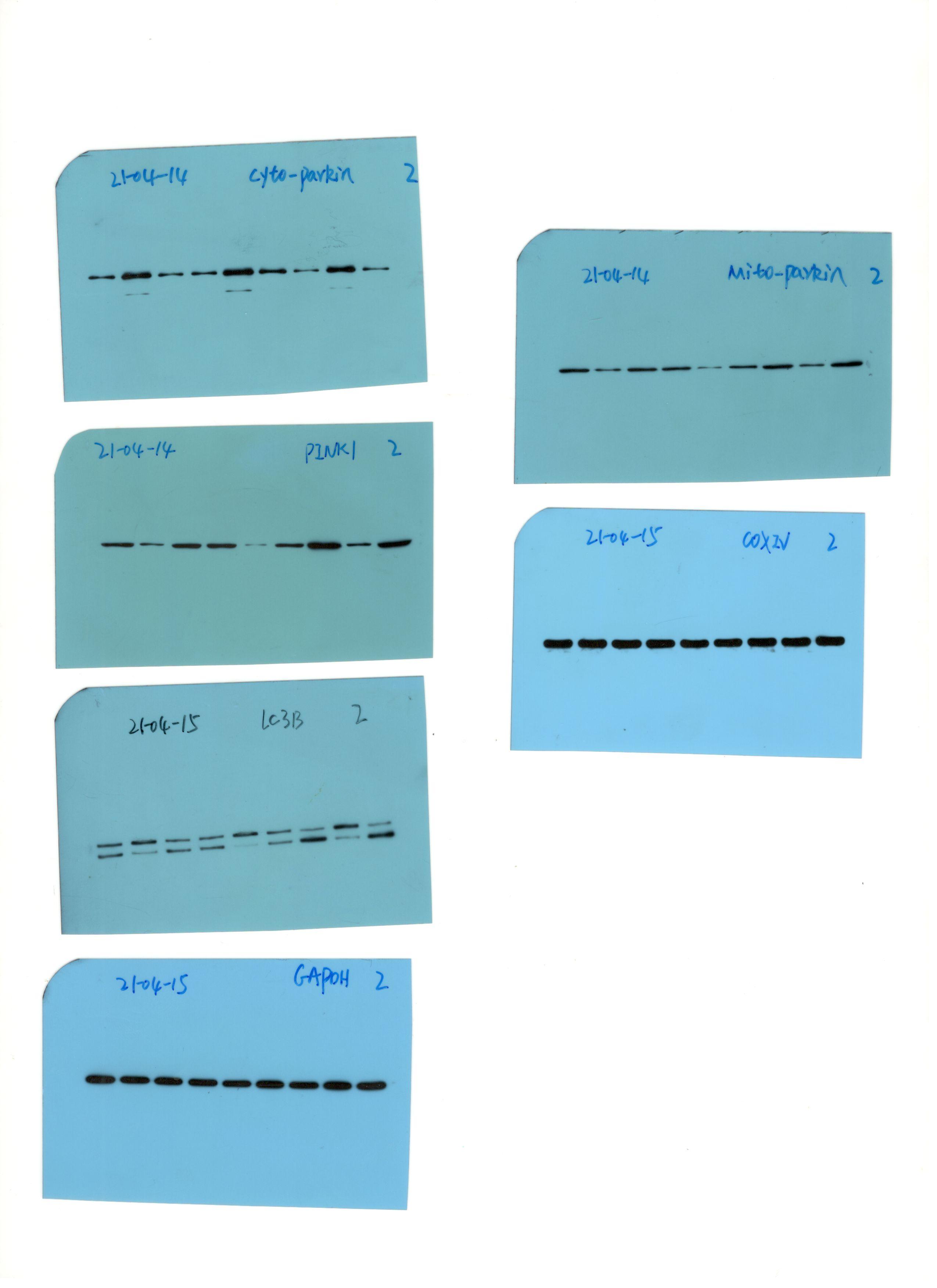


TET/Normal/Model/Benazpril/JQHF

TET/Normal/Model/Benazpril/JQHF

TET/Normal/Model/Benazpril/JQHF

TET/Normal/Model/Benazpril/JQHF

TET/Normal/Model/Benazpril/JQHF

TET/Normal/Model/Benazpril/JQHF

TET/Normal/Model/Benazpril/JQHF

LC3-I, 16KD

LC3-II, 14KD

GAPDH, 37KD

GAPDH, 37KD

COX IV, 17KD

PINK1, 63KD

Cyto-parkin, 52KD

Mito-parkin, 52KD

Supplementary Figure 2. Original blots for Figure 4.


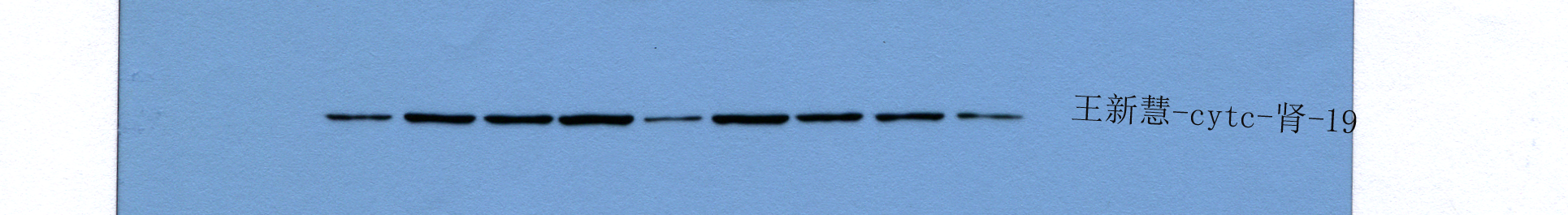


JQHF/ TET/ Benazpril/ Model/ Normal

Cytochrome c,14KD;

Used in Figure 4a


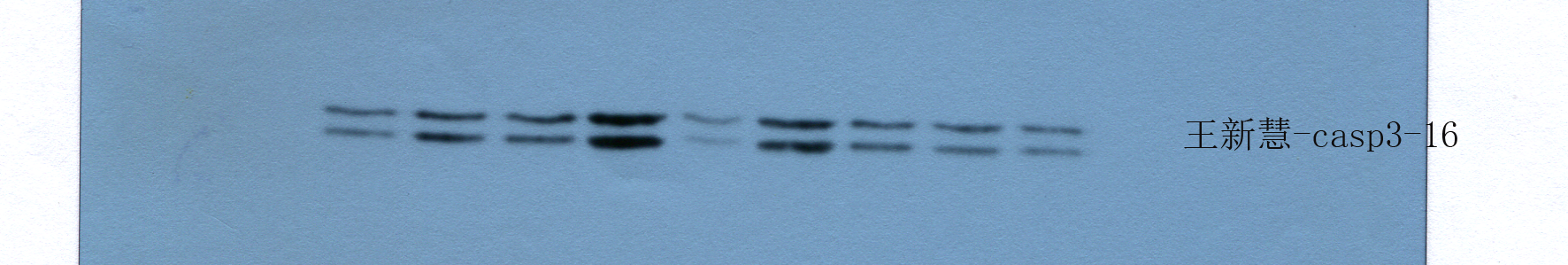


JQHF/ TET/ Benazpril/ Model/ Normal

Cleaved caspase-3, 17KD;

Used in Figure 4a

Cleaved caspase-3, 19KD

JQHF/ TET/ Benazpril/ Model/ Normal


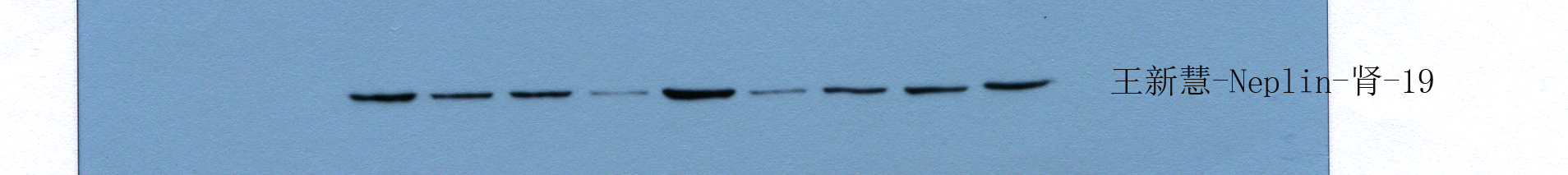


Nephrin,134KD;

Used in Figure 4e


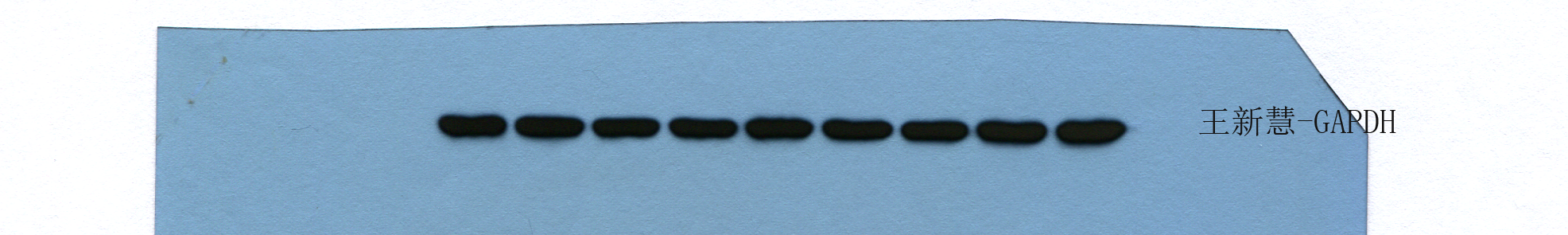


JQHF/ TET/ Benazpril/ Model/ Normal

GAPDH, 37KD;

Used in Figure 4a

JQHF/ TET/ Benazpril/ Model/ Normal


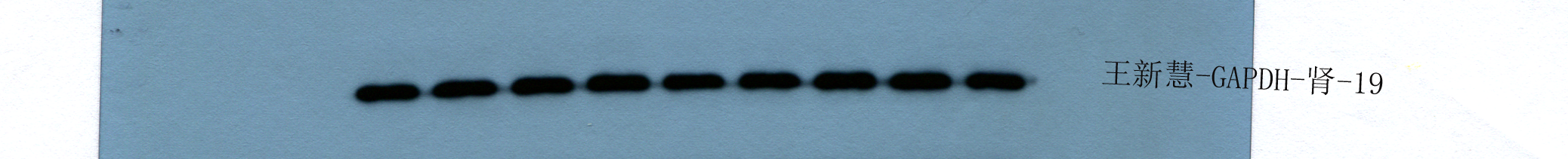


GAPDH, 37KD;

Used in Figure 4e;

Supplementary Figure 3. replicates performed blots for Figure 4.


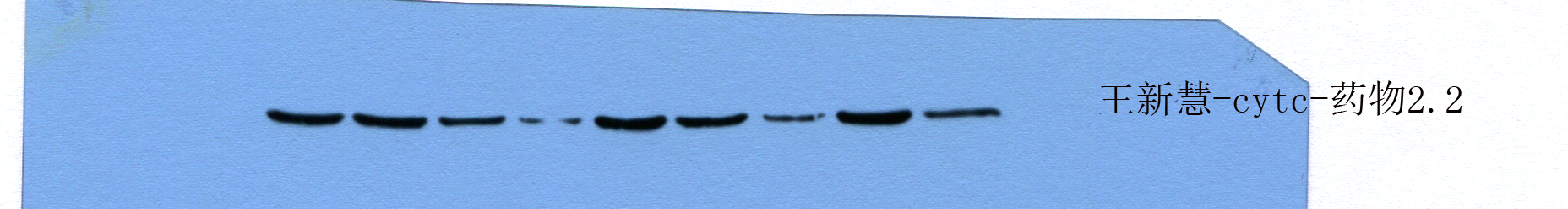

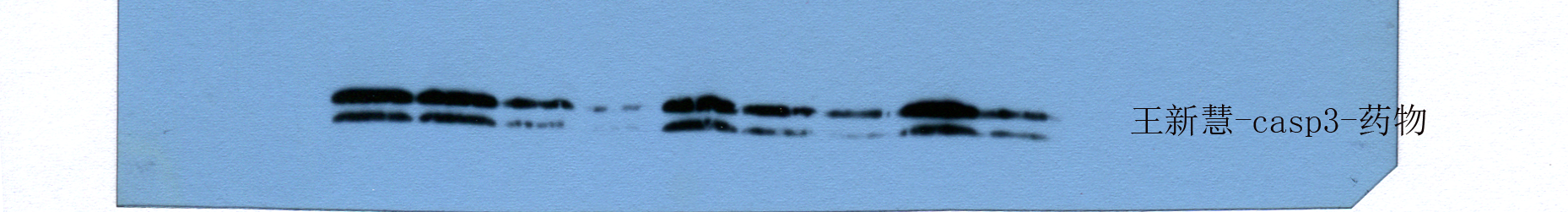

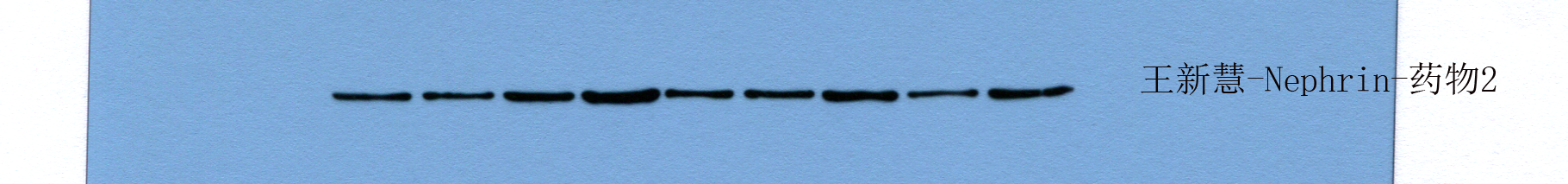

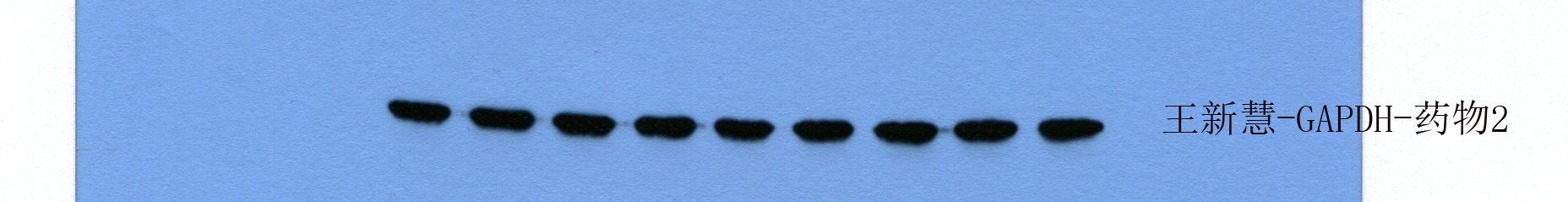


TET/Normal/Model/Benazpril/JQHF

TET/Normal/Model/Benazpril/JQHF

TET/Normal/Model/Benazpril/JQHF

TET/Normal/Model/Benazpril/JQHF

Cytochrome c,14KD;

replicates performed blots

Cleaved caspase-3, 17KD;

replicates performed blots

Nephrin,134KD;

replicates performed blots

GAPDH, 37KD;

replicates performed blots

JQHF/ TET/ Benazpril/ Model/Normal


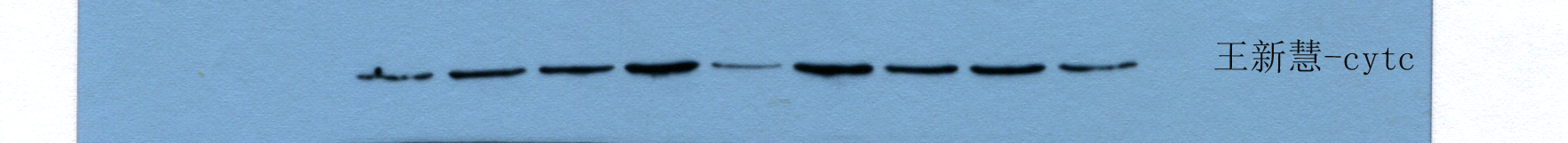


Cytochrome c,14KD;

replicates performed blots


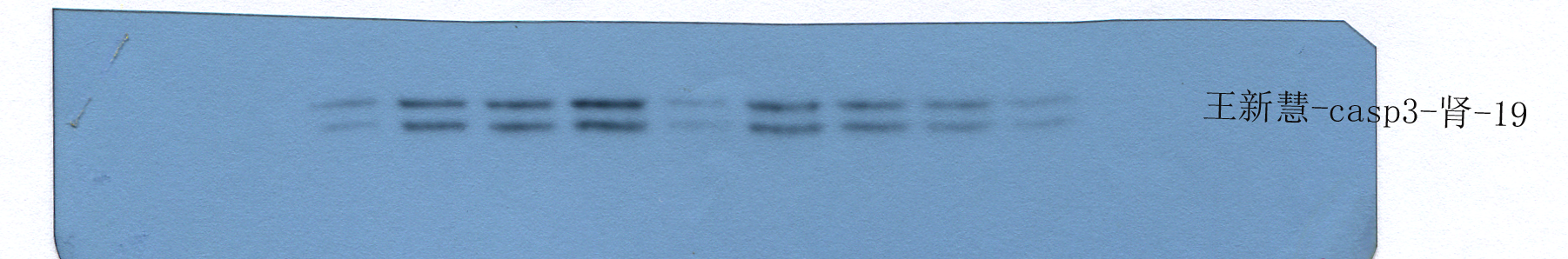


JQHF/ TET/ Benazpril/ Model/Normal

Cleaved caspase-3, 17KD;

replicates performed blots


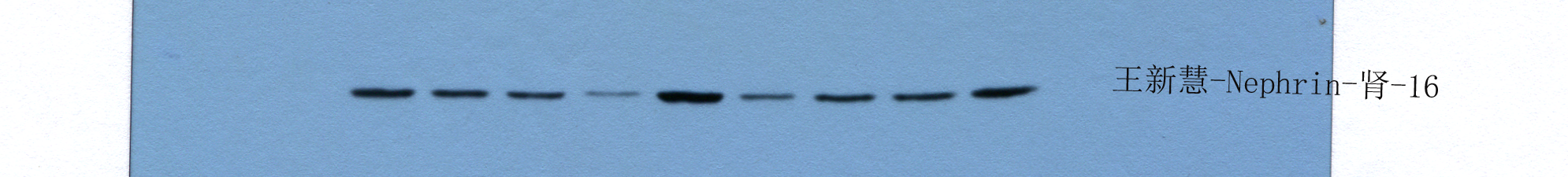

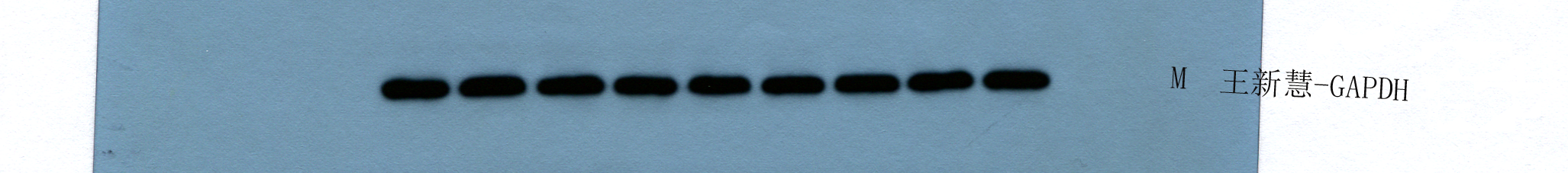


JQHF/ TET/ Benazpril/ Model/Normal

JQHF/ TET/ Benazpril/ Model/Normal

GAPDH, 37KD;

replicates performed blots

Nephrin,134KD;

replicates performed blots

Supplementary Figure 4. Original blots for Figure 4.


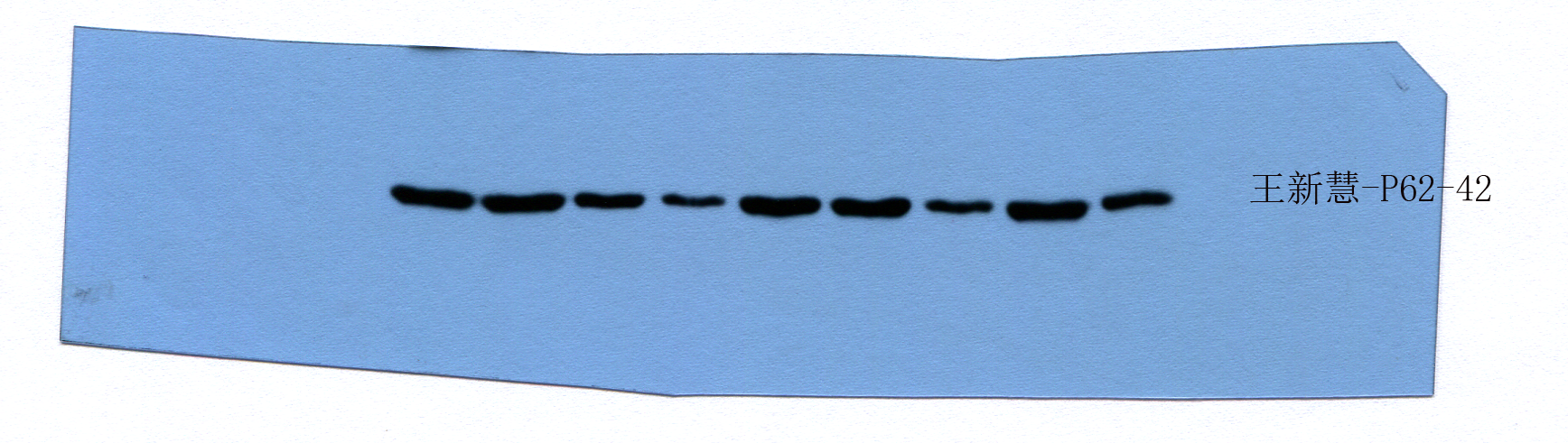


TET/Normal/Model/Benazpril/JQHF

SQSTM1/P62, 62KD;

Used in Figure 3a


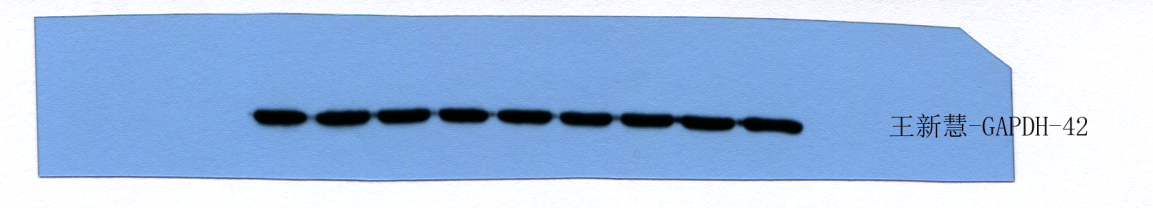


TET/Normal/Model/Benazpril/JQHF

GAPDH, 37KD;

replicates performed blots
